# Complex Vascular Reconstruction of an En Bloc Pediatric Kidney Damaged during Organ Procurement

**DOI:** 10.1155/2022/3242809

**Published:** 2022-06-16

**Authors:** Juliano Riella, Marina M. Tabbara, Phillipe Abreu, Javier Gonzalez, Gaetano Ciancio, José Maria Figueiro

**Affiliations:** ^1^Department of Surgery, University of Miami, Miami, FL, USA; ^2^Miami Transplant Institute, Miami, FL, USA; ^3^University of Miami Miller School of Medicine, Miami, FL, USA; ^4^Jackson Memorial Hospital, Miami, FL, USA; ^5^Department of Urology, Hospital General Universitario Gregorio Marañón, Madrid, Spain; ^6^Department of Urology, University of Miami, Miami, FL, USA

## Abstract

En bloc pediatric kidney (EBPK) allografts are a potential solution to expand the organ donor pool; however, EBPK transplantation has been traditionally considered suboptimal due to concerns of perioperative vascular and urologic complications. Accidental organ or vasculature injury during harvest is not uncommon; however, this does not necessarily mean that the organ should be discarded. Careful vascular reconstruction can be performed using donor vascular grafts, salvaging the organ without stenosis or thrombosis of the vessels. We report an extensive vascular reconstruction of the right renal artery, aorta, and inferior vena cava of a damaged EBPK allograft using a donor pediatric aorta vascular patch with the goal of avoiding postoperative vascular complications.

## 1. Introduction

Renal transplantation remains the best treatment option for patients with end-stage renal disease (ESRD). However, few ESRD patients on the waiting list receive a kidney transplant due to the wide gap between potential candidates and organ donors [1]. En bloc pediatric kidney (EBPK) transplantation represents a potential source to expand the kidney allograft donor pool [2].

Transplantation of small pediatric kidney allografts (donor age less than 3 years, donor weight less than 15 kg, and kidney size less than 6 cm) has been implemented more frequently over the years, but the increased risk of postoperative vascular and urologic complications, delayed graft function (DGF), or even primary graft nonfunction has made their utilization challenging in both pediatric and adult recipients [2–4]. Additionally, small pediatric renal allografts have been often reported to be damaged during the procurement procedure and their reconstruction and salvage are usually not attempted due to the greater risk of vascular complications [5]. Meticulous back-table reconstruction techniques have been previously highlighted and are the key in overcoming these issues [6].

We report the transplantation of an inadvertently injured EBPK in an adult recipient after extensive vascular reconstruction in the back-table operation. There was a damaged donor right renal artery reconstructed using a donor aortic vascular patch from the same pediatric donor.

## 2. Case Presentation

**Figure 1:**
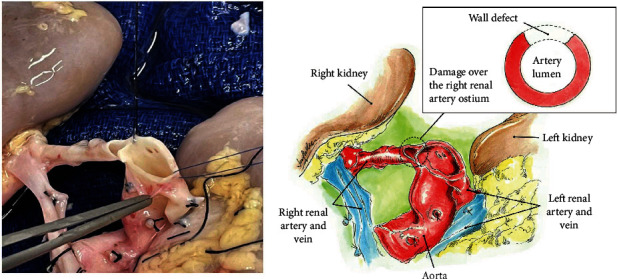
Aorta and damaged renal artery at the level of its takeoff.

**Figure 2:**
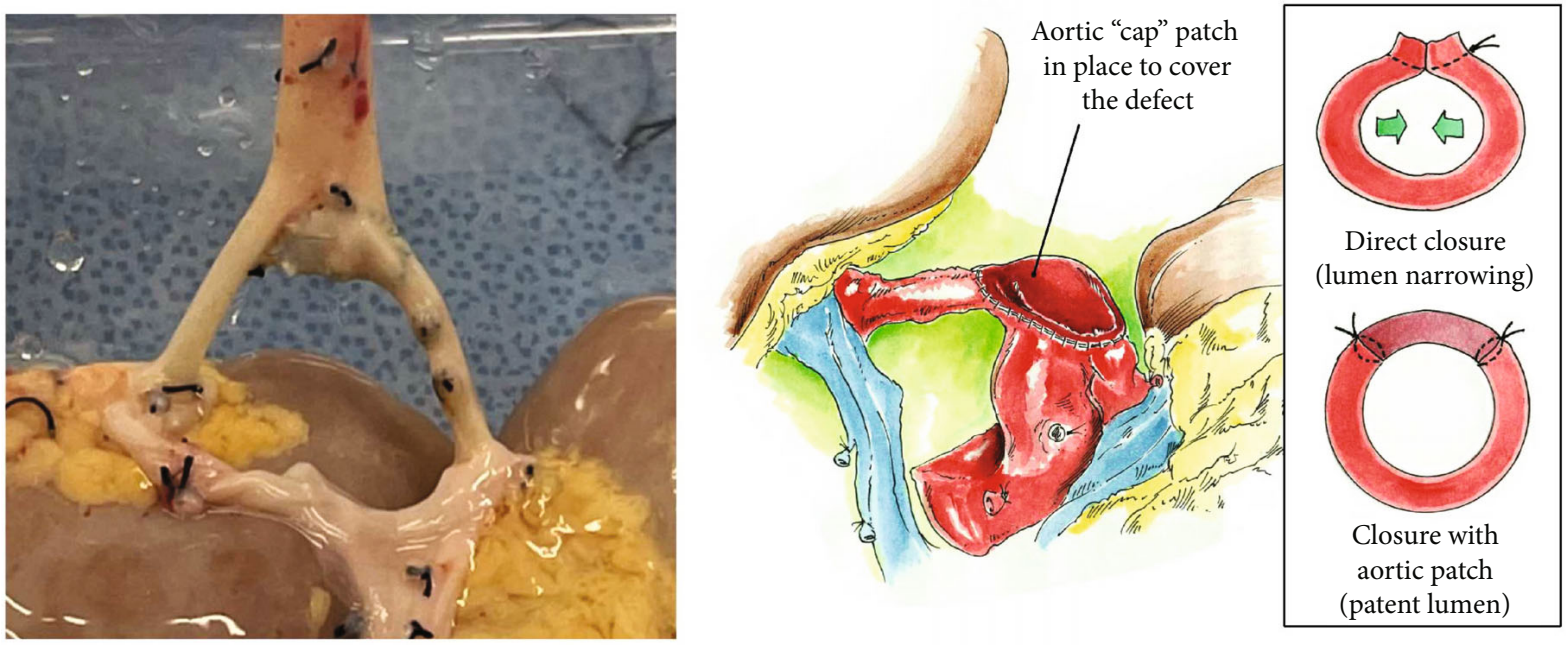
Reconstruction of the aorta and ostium of the right renal artery with the aortic patch from the same donor.

**Figure 3:**
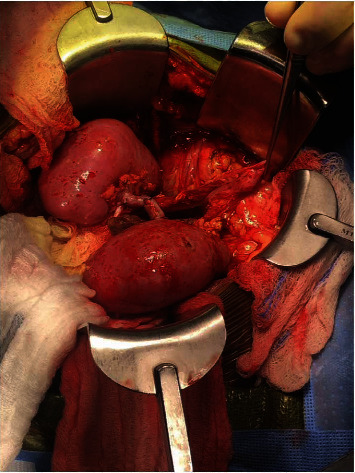
EBPK allograft placed in situ after transplantation. The vascular anastomoses between the aorta and IVC and recipient external iliac vessels have been finished. EBPK: en bloc pediatric kidneys; IVC: inferior vena cava.

The donor was a 13-month-old male deceased infant who died of anoxia secondary to unwitnessed cardiac arrest. His weight was 12.7 kg, with a terminal creatinine (Cr) level of 0.24 mg/dL and KDPI of 60%. The right and left kidneys both measured 5 cm long and 4 cm wide. During the kidney retrieval, the aorta and inferior vena cava (IVC) were divided cranially at the level of the takeoffs of both renal arteries and veins with damage to the takeoff of the right renal artery at its ostium (Figure 1). Since a direct closure of the proximal aorta vascular end would compromise the lumen of the damaged right renal artery and the opening of the left renal artery, same donor's aortic patch was used to repair the proximal end of the aorta without compromising the right and left renal arteries with 7-0 Prolene® running sutures (Figure 2). The kidneys were connected en bloc to the LifePort® renal preservation machine (RPM) and stored in hypothermia (2–4°C) using kidney perfusion solution (KPS-1®). Once placed on the RPM, the flow and resistance improved from 12 mL/min and 0.41 mmHg/mL/min to 66 mL/min and 0.27 mmHg/mL/min.  Figure 3 shows the EBPK allograft placed in situ after transplantation. The vascular anastomoses between the aorta and IVC and recipient external iliac vessels have been finished.

The recipient was a 28-year-old male who weighed 74 kg. He was on hemodialysis for 32 months. The recipient's external iliac vessels were dissected free, and the EBPK allograft was removed from the pulsatile perfusion machine and kept on ice.

The superior cap used for the aorta was fashioned to cover the right renal artery injury at the takeoff, preventing stenosis of the ostium. No increased velocities were identified on postoperative Doppler ultrasound. The vascular reconstruction was performed using the donor's own vessel grafts procured during multiorgan procurement that were not used for the primary transplants of other abdominal organs as the liver. These vessels are preserved in conservation solutions, flushed with heparin, and kept preserved at a temperature of 4°C.

The aorta and IVC were anastomosed end-to-side to the recipient's right external iliac vessels in a tension-free manner using running 6-0 Prolene® sutures. Finally, ureteroplasty was accomplished by side-to-side anastomosis of both distal ureters with 6-0 PDS interrupted suture and the Miami Transplant Institute extravesical ureteroneocystostomy with ureteral stent-free technique was performed onto the recipient bladder dome using 6-0 PDS interrupted sutures [7].

The warm ischemia time was 19 minutes, and the cold ischemia time was 1420 minutes. Surgical drainage or ureteral stent were not used. Postoperative Doppler ultrasound showed no collections in the perinephric space or signs of obstructive uropathy. Laminar blood flow and normal parameters (i.e., resistive index, peak systolic velocity and ratio, and *Z*-velocity) in both the external iliac and graft arteries were also recorded. The patient had an uneventful recovery showing a Cr level of 1.20 mg/dL at a 3-month follow-up.

## 3. Discussion

Since the first pediatric donor kidney transplantation in 1964, several advancements in surgical techniques, immunosuppression, and organ preservation technology have permitted the use of pediatric kidneys [8]. Recent evidence has confirmed that transplantation of pediatric kidneys offers a higher benefit in terms of survival as compared to remaining on dialysis [9].

There are several concerns in using EBPK, one of them is the increased risk of primary graft nonfunction, which could be secondary to a hyperfiltration injury. According to this theory described by Brenner [10], an adult patient receiving a small allograft could develop compensatory changes that can potentially cause progressive damage to the transplanted kidney. However, there is growing evidence that kidneys from young donors can adapt, increase its size, and improve glomerular filtration due to compensatory hypertrophy [11]. Primary nonfunction can also be immune related as early rejection can significantly cause nephron mass loss and affect the ability of the allograft to grow. So, in general, EBPK should be considered for nonsensitized recipients [12].

Another significant concern is related to vascular complications, Fananapazir et al. [9] found a significantly higher incidence of 9% thrombosis of arterial and venous origin in EBPK grafts. Al-Shraideh et al. [13] reported one (2.9%) thrombosis resulting in early graft loss in a group of 34 EBPK transplants. Graft renal artery stenosis is also reported with an incidence of 1 to 23% [14]. Doppler ultrasound appears to be less sensitive to diagnose stenosis in this patient population. Bent et al. [15] report that of 25 EBPK recipients with a Doppler ultrasound of donor aorta or renal artery velocities greater than 300 cm/s, only 2 patients (4%) had angiographically significant stenosis.

In order to reduce the risk of these vascular complications, we describe the use of cap-shaped patches from the same donor as a conduit in both proximal ends of the allograft aorta and IVC and at the distal allograft vascular end, providing increased length to reduce excessive traction and subsequent thrombosis and/or stenosis [16, 17]. The use of elongation patches and conduits proves to be a useful and safe method for decreasing the incidence of vascular-related complications in transplantation [3].

In regard to urologic complications, a retrospective study by Fananapazir et al. [18] demonstrated an incidence of ureteral complications of up to 9.8% after EBPK. The stricture was the most common followed by urinary leak. Despite this, half of the cases were managed nonoperatively without impact on the patient or graft survival; thus, this should not prevent the utilization of EBPK for appropriate recipients.

The continuous shortage of organ offers and the increase in the kidney transplant waiting list demand for the more aggressive use of extended criteria donors including EBPK [13, 19]. Growing literature has been demonstrating its feasibility and safety, and despite procurement-related vascular injuries, these organs can still be salvaged using meticulous back-table vascular reconstruction techniques and obtaining good allograft function without increased risk of vascular and/or urological complications.

## Data Availability

The raw data supporting the conclusion of this article will be made available by the authors, without undue reservation.
